# Salivary responses to chewing mastic resin and parafilm and inhalation of mastic- and α-pinene-derived volatiles in healthy young adults

**DOI:** 10.3389/fdmed.2026.1888616

**Published:** 2026-08-04

**Authors:** Zainab Assy, Wiktoria Potocka, Chadidzja Albogatjiew, Asiye Özcan, Floris J. Bikker, Marja L. Laine

**Affiliations:** 1 Department of Oral Biochemistry, Academic Centre for Dentistry Amsterdam, University of Amsterdam and VU University Amsterdam, Amsterdam, Netherlands; 2 Department of Periodontology, Academic Centre for Dentistry Amsterdam, University of Amsterdam and VU University Amsterdam, Amsterdam, Netherlands

**Keywords:** flow rate, mastic, pH, saliva, terpenes

## Abstract

Salivary secretion is essential for maintaining oral homeostasis, and its modulation by sensory stimuli provides insight into salivary gland function. Mastic resin, obtained from the *Pistacia lentiscus* tree, is rich in terpenes such as α-pinene, which may influence salivary responses through gustatory, olfactory, or mechanosensory pathways. In addition, α-pinene may modulate salivary secretion through cholinergic pathways and may therefore have potential as a natural sialagogue. We aimed to evaluate how healthy young adults respond to masticatory and olfactory stimulation by assessing the changes in salivary flow rate and pH following exposure to mastic resin, a tasteless chewing control, and volatiles of mastic oil or α-pinene. The salivary flow and pH were measured in healthy individuals (*n* = 30) before and after chewing mastic resin or parafilm (control), or after inhalation of mastic or α-pinene oil volatiles delivered via nasal inhalers. Both masticatory stimuli increased salivary flow (*p* < 0.0001). Chewing mastic resin induced a greater increase in salivary flow than chewing parafilm (*p* < 0.0001). Olfactory exposure to mastic oil and α-pinene also elicited significant increases in salivary flow (*p* ≤ 0.01). Furthermore, pH increased after chewing mastic resin and parafilm (*p* < 0.0001) and α-pinene inhalation (*p* < 0.05), but not after inhalation of mastic volatiles. In conclusion, healthy young adults demonstrated clear salivary responses to both chewing and odour stimuli derived from *P. lentiscus.* The findings demonstrate that mastic resin and its volatile components can modulate salivary secretion through multiple sensory pathways, and they warrant further investigation of mastic-derived stimuli as potential modulators of salivary secretion, especially in individuals with dry mouth.

## 1 Introduction

Saliva plays an important role in maintaining oral homeostasis and contributes to lubrication, buffering, digestion, and sensory perception. Salivary flow rates are dynamically regulated by sensory stimulation, including masticatory activity, gustatory inputs, and olfactory cues (1). Characterising how healthy young adults respond to such stimuli provides important insight into the physiological mechanisms governing salivary gland function under everyday sensory conditions.

Mastic resin, a natural secretion of the *Pistacia lentiscus* tree, represents an interesting stimulus due to its combined mechanical and chemosensory properties. *Pistacia* spp trees can be found in the Mediterranean region, in particular Greece, Morocco, and Algeria, but also in the Middle East, in Iran and Iraq (2, 3). The mastic resin is rich in volatile terpenes, α-pinene being the most abundant (4).

Terpenes have been extensively investigated for their potential benefits in oral health, demonstrating antimicrobial and antiseptic properties. In addition, these compounds have been implicated in the management of dry mouth. Plant-derived products containing terpenes—such as those obtained from ginger, mallow, bergamot, and liquorice—have been reported to stimulate salivary flow (5). These products are rich in monoterpenes and their derivatives, including α-pinene, eugenol, geraniol, eucalyptol, and linalool, which are hypothesized to contribute to their observed sialagogic effects (5). Despite these findings, the extent to which these volatile compounds enhance salivary secretion at the population level remains unclear. In particular, the proportion of individuals exhibiting a measurable sialagogic response to terpene-rich formulations has not yet been systematically evaluated.

α-Pinene has been identified as a mild acetylcholinesterase (AChE) inhibitor (6, 7). By modulating acetylcholine turnover, α-pinene may enhance parasympathetic activity, which is closely linked to salivary secretion via the cholinergic pathway (8). In addition, both chewing and exposure to odorants such as α-pinene may activate neural circuits associated with salivary reflexes, suggesting a possible modulatory effect on salivary gland activity (1).

Traditionally, mastic resin has been used as a natural flavouring agent, a thickening and stabilising agent, and as a chewing gum (9–11). However, little is known about salivary responses in healthy young adults to the combined mechanical and chemosensory stimulation from chewing mastic resin, or to olfactory stimulation from mastic-derived volatiles. Therefore, the present study aimed to characterise salivary flow and pH responses to these stimuli. The main aim of the current study was to evaluate how healthy young adults respond to masticatory and olfactory stimulation by assessing the changes in salivary flow rate and pH following exposure to mastic resin, a tasteless chewing control, and volatiles of mastic oil or α-pinene.

## 2 Materials and methods

### 2.1 Participants

Thirty students from the Academic Centre for Dentistry Amsterdam (ACTA), the Netherlands, were recruited for this study. All procedures were performed in compliance with the relevant laws and institutional guidelines and were approved by the local Ethical Committee of ACTA with protocol no. 2018003. The participants gave their written informed consent and were asked to complete a health questionnaire. Alongside the health questionnaire, participants were asked to complete the Xerostomia Inventory (XI) (12). Healthy individuals (with no active caries or mucosal lesions) over the age of 18 years were included. Exclusion criteria were (self-reported): periodontitis, gingivitis, halitosis, medication affecting saliva secretion, radiation in the head and neck area, and an upper respiratory infection.

### 2.2 Mastic resin and nasal inhalers preparation

Mastic resin (pH 7.4; origin: Iran, gifted by Mrs Rosemarie Leusink, Doorn, the Netherlands) was dehydrated in an incubator at 40 °C. Afterwards, the resin was kneaded until homogeneous. A total of 0.39 g of resin was portioned and rolled into a smooth ball using gloved hands. The balls were air-dried until the outer surface hardened. The balls were packed in plastic wrappers and stored in a dark, dry environment.

Nasal inhalers were prepared using a 2.5% solution of either α-pinene oil (CAS 80-56-8, Cibiday, Rotterdam, the Netherlands) or mastic resin oil (CAS 61789-92-2, Anemos, Chios, Greece) in refined olive oil (food-grade, commercially available; Amsterdam, the Netherlands). Subsequently, 1 mL of the solution was added to the cotton insert of the nasal inhaler (YBMC, Raalte, the Netherlands). α-Pinene oil consisted of 99.7% α-pinene, and mastic resin oil consisted of 70%–85% α-pinene, 10%–18% β-myrcene, 2%–3.5% β-pinene, 0.5%–1.5% limonene, 0.3%–1.2% β-caryophyllene, and 0.2%–1% camphene. To eliminate possible effects of refined olive oil on salivary responses, placebo nasal inhalers were prepared containing 1 mL of refined olive oil.

### 2.3 Saliva collection protocol

Participants were instructed not to smoke, eat or drink, use chewing gum, exercise, brush their teeth, or wear scented perfume or cosmetics for at least 2 h prior to saliva collection. Before collection, participants rinsed their mouths with tap water.

For unstimulated whole saliva (UWS), participants were asked to pool saliva in the floor of the mouth and passively drool into a 30 mL transparent polypropylene cup (Boomlab, Meppel, the Netherlands) after 5 min (13).

Chewing-stimulated whole saliva (P-CH-SWS) was collected by asking participants to chew 0.39 g of Parafilm (Bemis Europe, Braine L'Alleud, Belgium) and passively drool into the tube every 30 s over a 5-minute period. Mastic chewing-stimulated whole saliva (M-CH-SWS) was collected in the same manner while participants chewed 0.39 g of mastic resin.

The transparent polypropylene cups were measured before and after saliva collection on an analytical weighing scale (Sartorius, Göttingen, Germany). For salivary flow measurement, 1 g was considered as 1 mL. The pH was measured with an electronic pH meter (Radiometer, Copenhagen, Denmark).

For scent-stimulated whole saliva collection using mastic oil or α-pinene oil, participants used nasal inhalers held underneath the nose (0.5–1 cm below the septum, at a 60° angle from the face). They performed one active inhalation in each nostril every 30 s (10 inhalations in total), followed by passive drooling into the tube after 5 min (14). The same protocol was applied to assess the placebo effect using refined olive oil only.

Saliva sampling was conducted in the morning (9:00–12:00 a.m.) to minimise circadian variation (15, 16). Each type of stimulation was administered on a different day. Participants received the stimulating compounds in random order and were unaware of which compounds they were receiving (single-blind design). On each study day, UWS was collected first, followed by one of the following: parafilm chewing (P-CH), mastic resin chewing (M-CH), α-pinene scent stimulation, or mastic scent stimulation.

### 2.4 Statistical analysis

The statistical analysis was carried out using GraphPad Prism (Dotmatics, Massachusetts, USA). The change in salivary flow was calculated: Δ = SWS—UWS (mL/min). Data normality was assessed using the Shapiro–Wilk test (*p* < 0.05). Changes in salivary flow and pH after stimulation with parafilm, mastic, and α-pinene were analysed using the Wilcoxon signed-rank test. The number of responders was determined for the parafilm chewing, mastic chewing, and scent stimulation groups. Previous research has shown that an increase or decrease of 45% from the initial baseline value could be considered as normal salivary variation (17). Therefore, for exploratory purposes, participants showing an increase of ≥50% relative to baseline UWS values were classified as responders. Values are reported as mean ± SD (normally distributed data) unless stated otherwise. *P*-values <0.05 were considered statistically significant.

## 3 Results

Thirty individuals participated in this study. Of these, 22 (73%) were female, and 8 (27%) were male, with a mean age of 21.6 ± 3.3 years. The mean XI score was 19.77 ± 5.64. Furthermore, five (16.7%) participants used medication, 17 (56.7%) reported experiencing stress, and three (10%) reported having pre-existing diseases that did not affect salivation.

The scent of refined olive oil (placebo) did not significantly affect salivary secretion (*p* > 0.05, *n* = 9). Median UWS flow was 0.26 mL/min (0.20–0.35 mL/min), compared with 0.20 mL/min (0.14–0.36 mL/min) after placebo inhalation.

**Figure 1:**
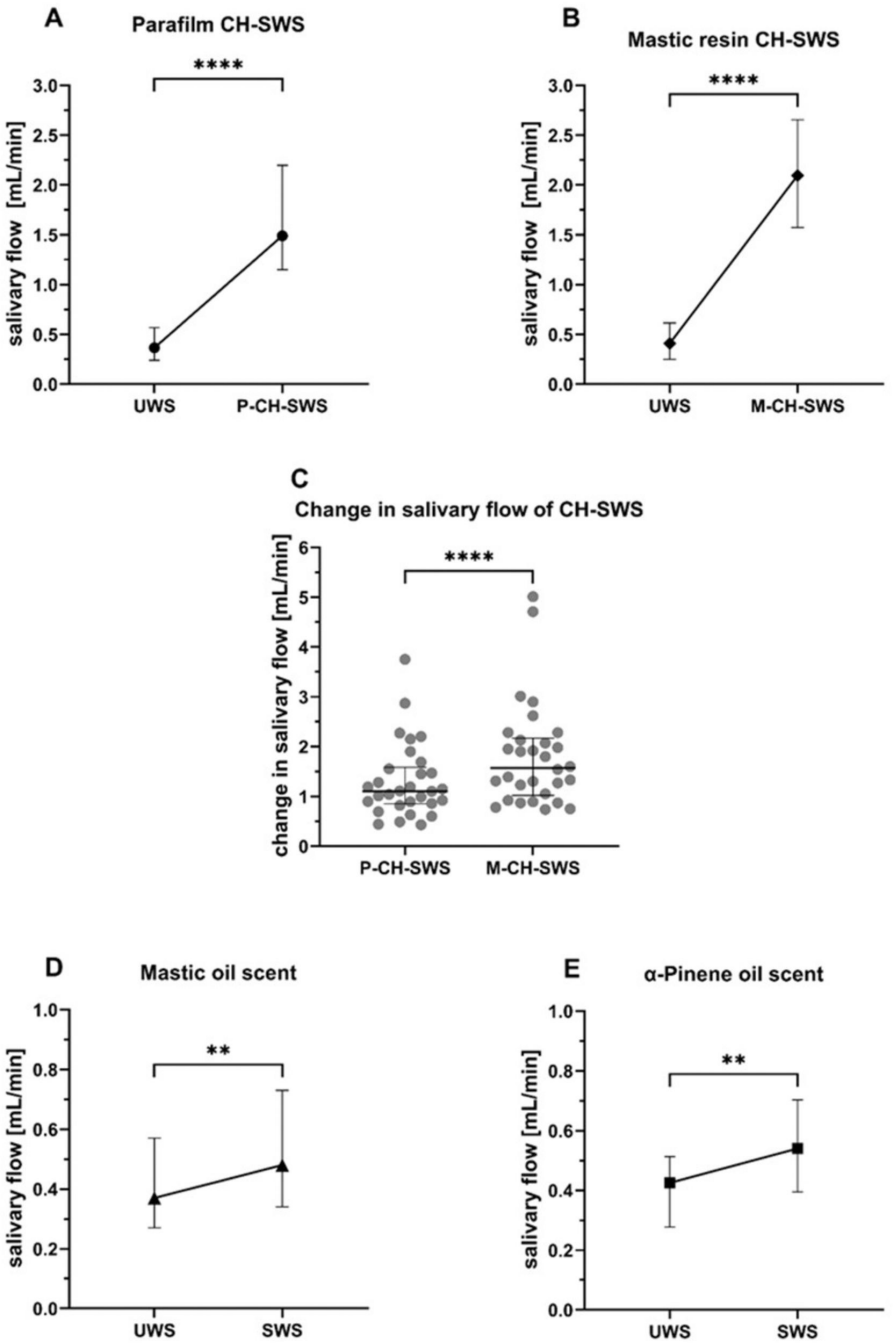
Salivary flow (mL/min) before (UWS) and after chewing parafilm (P-CH-SWS) **(A)** or mastic resin (M-CH-SWS) **(B)** in healthy participants (*n* = 30). Change in salivary flow after chewing parafilm (P-CH-SWS) or mastic resin (M-CH-SWS) **(C)**. Salivary flow (mL/min) before (UWS) and after stimulation (SWS) with **(D)** mastic oil scent (*n* = 27), **(E)** α-pinene scent (*n* = 28). Values are expressed as median (IQR). Wilcoxon signed-rank test. **p ≤ 0.01, ****p < 0.0001.

There was a significant increase in salivary flow between UWS [0.37 (0.24–0.57) mL/min] and P-CH-SWS [1.49 (1.15–2.20) mL/min, *p* < 0.0001] (Figure 1A). A similar increase in salivary flow was observed after chewing mastic resin [median (IQR), UWS 0.41 (0.25–0.62) mL/min vs. M-CH-SWS 2.10 (1.57–2.66) mL/min, *p* < 0.0001] (Figure 1B). The increase in salivary flow was greater after chewing the mastic resin [median (IQR), Δ-M-CH-SWS 1.57 (1.02, 2.17) mL/min], than after chewing parafilm [ΔP-CH-SWS 1.11 (0.85, 1.59) mL/min, *p* < 0.0001] (Figure 1C).

Exposure to mastic oil scent increased salivary flow [UWS 0.37 (0.27–0.57) mL/min, SWS 0.48 (0.34–0.73) mL/min, *p* ≤ 0.01]. A similar increase was observed for α-pinene oil scent [UWS 0.43 (0.28–0.51) mL/min, SWS 0.54 (0.40–0.70) mL/min, *p* ≤ 0.01] (Figures 1D,E). No difference was observed in the change in salivary flow when comparing mastic oil scent and α-pinene oil scent stimulation (data not shown).

All participants (100%) showed a positive response to parafilm chewing and mastic resin chewing. In contrast, 30% (8 out of 27) of participants showed a positive response to olfactory stimulation with mastic oil, and 29% (8 out of 28) showed a positive response to α-pinene oil. The pH increased significantly from UWS to P-CH-SWS [6.80 (6.68–7.00) to 7.20 (7.20–7.33), *p* < 0.0001] (Table 1). Furthermore, salivary pH increased significantly after chewing mastic resin [UWS 6.90 (6.70–7.03) to 7.30 (7.20–7.40), *p* < 0.0001], and after stimulation with α-pinene oil scent [UWS 6.80 (6.53–7.00) to 6.90 (6.80–7.08), *p* < 0.05], but not after stimulation with mastic oil scent.

**Table 1:** Salivary pH before (UWS) and after stimulation (SWS) with chewing parafilm, chewing mastic resin, mastic oil scent and α-pinene oil scent in healthy participants.

Stimulation type (*n* participants)	UWS (pH)	SWS (pH)	Statistical significance
Chewing parafilm (*n* = 30)	6.80 (6.68–7.00)	7.20 (7.20–7.33)	*p* < 0.0001
Chewing mastic resin (*n* = 30)	6.90 (6.70–7.03)	7.30 (7.20–7.40)	*p* < 0.0001
Mastic oil scent (*n* = 27)	6.90 (6.60–7.10)	6.90 (6.70–7.10)	ns
α-Pinene oil scent (*n* = 28)	6.80 (6.53–7.00)	6.90 (6.80–7.08)	*p* < 0.05

Values are expressed as median (IQR). Wilcoxon signed-rank test. ns, not significant.

## 4 Discussion

This study compared the effects of chewing mastic resin and parafilm as a control on salivary flow and pH. Additionally, the effects of inhaling mastic oil and α-pinene oil volatiles, delivered via nasal inhalers, on salivary flow and pH were evaluated in healthy individuals. The proportion of responders to each intervention was also determined.

Chewing mastic resin and olfactory stimulation with terpene oils resulted in an increase in salivary flow. Chewing mastic resin resulted in higher salivary flow than chewing parafilm only, which is a part of standard salivary tests in a clinical setting, alongside citric acid stimulation (16, 18). This suggests that, next to mechanical stimulation, there could be an additional effect of the terpene compounds found in mastic resin on salivary flow. There was no difference between the stimulation with mastic oil scent and α-pinene scent.

Both parafilm and mastic resin chewing resulted in an increase in pH. Furthermore, salivary pH increased significantly after the olfactory stimulation with α-pinene oil scent, but this effect was not observed after the stimulation with mastic oil scent. Salivary pH is a key determinant in the dynamic equilibrium between tooth demineralization and remineralization processes. An increase in pH is essential for buffering due to the availability of bicarbonate, phosphate, and urea in saliva (19, 20). The observed increase in pH following α-pinene stimulation suggests that secretion from the major salivary glands, including both fluid and electrolyte components, remains within physiological limits (19, 20). Interestingly, olfactory stimulation with mastic oil did not result in a significant increase in salivary pH, whereas exposure to α-pinene alone elicited a measurable elevation. This finding was unexpected, given that mastic oil is composed predominantly of α-pinene (approximately 70%–85%) (21). Based on its chemical composition, a similar pH response would have been anticipated following mastic oil stimulation. However, mastic oil contains several terpene constituents besides α-pinene, such as β-myrcene, β-pinene, limonene, β-caryophyllene, and camphene. It is conceivable that interactions among these volatile compounds may alter olfactory receptor activation and downstream autonomic responses involved in salivary gland stimulation (1, 8). Furthermore, terpene mixtures may differentially modulate transient receptor potential (TRP) channels, which have previously been implicated in salivary secretion and saliva composition (22–24). Such interactions could potentially attenuate the pH response observed with α-pinene alone.

Previous studies from our research group have demonstrated that both mastic resin in its natural form and mastic- or α-pinene-derived volatiles delivered via nasal inhalers possess sialagogic properties in healthy individuals and patients with dry mouth (25, 26). In line with these findings, the mechanisms underlying the observed salivary stimulation may involve multiple pathways regulating salivary gland activity (14, 15, 22–26), including AChE inhibition and interactions with TRP channels. However, olfactory stimulation appeared less effective than chewing stimulation. Chewing typically induces a three- to fivefold increase in salivary flow compared with unstimulated flow rates (1), which may explain why a larger proportion of participants responded positively to chewing than to odour stimulation. These findings may contribute to the development of novel interventions for dry-mouth management.

The proportion of positive responders was lower in the olfactory stimulation group than in the chewing group. These differences may be explained by the physiological contribution of the parotid gland, which accounts for up to 60% of total salivary flow under stimulated conditions, particularly in response to mastication and gustatory stimuli (20). Chewing mastic resin likely induces a stronger parotid response due to the combined effects of mechanical stimulation and taste, including the characteristic pine-like flavour, which may further enhance glandular activation. In addition, retronasal olfactory stimulation may play a role during mastication. Volatile compounds released during chewing reach the olfactory receptors via the retronasal pathway, potentially augmenting salivary secretion and influencing salivary composition, including pH. In contrast, orthonasal stimulation follows distinct neural pathways (25, 26). The observed effects may be mediated through mechanisms such as modulation of parasympathetic activity via olfactory–autonomic interactions and/or through inhibition of acetylcholinesterase at the synaptic level, thereby enhancing cholinergic signalling to salivary acinar cells (25, 26). However, these mechanisms remain speculative and require further investigation.

It should also be noted that a subset of participants in the present study reported experiencing stress, which is known to suppress parasympathetic-driven salivary secretion. This factor may have contributed to the reduced number of positive responders in the olfactory stimulation group. Although XI-scores and UWS flow rates were within normal ranges, future studies should aim to minimise confounding factors such as stress. Inclusion of participants under controlled physiological conditions would allow for a more accurate assessment of the true effects of olfactory stimulation on salivary function.

This study has several limitations, including the relatively small cohort size and the inclusion of a specific demographic consisting of young adults enrolled in dental education. Consequently, the study population may not be representative of the general population or of patients with dry mouth, who are typically older. Furthermore, individuals undergoing dental education may demonstrate greater awareness of oral health practices than the general population. Nevertheless, previous studies have shown that the sialagogic effects of mastic-derived stimuli are also present in older age groups (30–40 years old) (13, 22) and in patients diagnosed with dry mouth (18). Another limitation of the present study is the exclusive focus on objective parameters, without assessment of participants' subjective experiences following chewing or olfactory stimulation. While baseline xerostomia was evaluated using the XI questionnaire, no post-intervention subjective outcomes were collected. In addition, the perceived pleasantness or acceptability of the stimuli was not assessed. Incorporating subjective measures is important, as patient perceptions and experiences are critical to the evaluation of therapeutic interventions. Patient-reported outcome measures (PROMs) are increasingly recognised as key endpoints in clinical research and may, in some cases, serve as primary outcomes (27). Therefore, future studies should include both objective and subjective assessments to provide a more comprehensive evaluation of the effects of scent-based stimulation on salivary function and overall patient experience.

In conclusion, this study demonstrated that healthy individuals exhibit measurable salivary responses to both chewing mastic resin and olfactory stimulation with mastic- and α-pinene-derived volatiles. Following validation in patients with dry mouth, these findings may contribute to the development of novel strategies for dry-mouth management.

## Data Availability

The original contributions presented in the study are included in the article/Supplementary Material, further inquiries can be directed to the corresponding author.
